# A SCN9A gene-encoded dorsal root ganglia sodium channel polymorphism associated with severe fibromyalgia

**DOI:** 10.1186/1471-2474-13-23

**Published:** 2012-02-20

**Authors:** Gilberto Vargas-Alarcon, Edith Alvarez-Leon, Jose-Manuel Fragoso, Angelica Vargas, Aline Martinez, Maite Vallejo, Manuel Martinez-Lavin

**Affiliations:** 1 Department of Molecular Biology, National Institute of Cardiology Ignacio Chavez, Mexico city, Mexico; 2 Department of Rheumatology, National Institute of Cardiology Ignacio Chavez, Mexico city, Mexico; 3 Department of Sociomedical Investigation, National Institute of Cardiology Ignacio Chavez, Mexico city, Mexico

**Keywords:** Fibromyalgia, SCN9A, Sodium channels, Sympathetic pain, Dorsal root ganglia, Sympathetic nervous system, Autonomic nervous system, Neuropathic pain, Sodium channelopathy

## Abstract

**Background:**

A consistent line of investigation suggests that autonomic nervous system dysfunction may explain the multi-system features of fibromyalgia (FM); and that FM is a sympathetically maintained neuropathic pain syndrome. Dorsal root ganglia (DRG) are key sympathetic-nociceptive short-circuit sites. Sodium channels located in DRG (particularly Nav1.7) act as molecular gatekeepers for pain detection. Nav1.7 is encoded in gene SCN9A of chromosome 2q24.3 and is predominantly expressed in the DRG pain-sensing neurons and sympathetic ganglia neurons. Several SCN9A sodium channelopathies have been recognized as the cause of rare painful dysautonomic syndromes such as paroxysmal extreme pain disorder and primary erythromelalgia. The aim of this study was to search for an association between fibromyalgia and several SCN9A sodium channels gene polymorphisms.

**Methods:**

We studied 73 Mexican women suffering from FM and 48 age-matched women who considered themselves healthy. All participants filled out the Fibromyalgia Impact Questionnaire (FIQ). Genomic DNA from whole blood containing EDTA was extracted by standard techniques. The following SCN9A single-nucleotide polymorphisms (SNP) were determined by 5' exonuclease TaqMan assays: rs4371369; rs4387806; rs4453709; rs4597545; rs6746030; rs6754031; rs7607967; rs12620053; rs12994338; and rs13017637.

**Results:**

The frequency of the rs6754031 polymorphism was significantly different in both groups (*P *= 0.036) mostly due to an absence of the GG genotype in controls. Interestingly; patients with this rs6754031 GG genotype had higher FIQ scores (median = 80; percentile 25/75 = 69/88) than patients with the GT genotype (median = 63; percentile 25/75 = 58/73; *P *= 0.002) and the TT genotype (median = 71; percentile 25/75 = 64/77; *P *= 0.001).

**Conclusion:**

In this ethnic group; a disabling form of FM is associated to a particular SCN9A sodium channel gene variant. These preliminary results raise the possibility that some patients with severe FM may have a dorsal root ganglia sodium channelopathy.

## Background

The background information leading to the present investigation has been already published as a hypothesis [1] and is summarized here: A consistent line of investigation suggests that autonomic nervous system dysfunction may explain the multi-system features of fibromyalgia (FM); and that FM is a sympathetically maintained neuropathic pain syndrome. Trauma and infection are two recognized FM triggers. In animal models; trauma or infection can induce phenotypic changes in the dorsal root ganglia (DRG) leading to a persistent sympathetically-maintained pain state [1].

DRG are nodules that lie along the spinal column. They play a key role in pain perception. DRG house the cell bodies of sensory neurons; the dendrites of which are located in the skin; muscles; tendons; joints; and internal organs. DRG act also as viral sanctuaries. Under normal circumstances; DRG have scant sympathetic innervation. Nevertheless; trauma and/or infection trigger sympathetic sprouting within DRG via nerve growth factor over-expression. Such aberrant neuroplasticity enables catecholamines and sympathetic traffic to induce sensory neuron firing. Sodium channels play a pivotal role in this hyperexcitability. These mechanisms are the basis of the sympathetically-maintained pain concept [1].

Sodium channels located in DRG act as molecular gatekeepers of pain detection at peripheral nociceptors. Nine sodium channel subunits have been identified (Nav1.1-Nav1.9); each with a unique central and peripheral nervous system distribution. An isoform (Nav1.7) encoded in gene SCN9A of chromosome 2q24.3 is predominantly expressed in the DRG pain-sensing neurons and sympathetic ganglia neurons [2]. Different Nav1.7 mutations induce electrical hyperactivity of sensory neurons in DRG and; at the same time; produces hypo-reactivity of sympathetic ganglia neurons [3-5]. Several sodium "channelopathies" have been associated to rare painful dysautonomic syndromes such as primary erythromelalgia and paroxysmal extreme pain disorder (formerly familial rectal pain syndrome). Moreover; a nucleotide polymorphism in the SCN9A gene (rs6746030) was related to pain perception intensity in individuals with different painful diseases [6]. This polymorphism makes DRG neurons hyperexcitable [7].

This information lead us to the present investigation whose objective was to search for an association between several SCN9A sodium channels gene polymorphisms and fibromyalgia.

## Methods

We studied 73 Mexican women suffering from FM according to the 1990 American College of Rheumatology criteria. These patients were referred from different private rheumatology practices in Mexico. As controls; we studied 48 Mexican women who considered themselves to be healthy and who denied having chronic pain. Patients and controls were matched by age. Mexicans have diverse ethnicity. The great majority have European and Native American background. "Mestizo" is the term used to name this mixed heritage. All individuals included in the study were born in Mexico and were catalogued as Mestizo. No further genealogical characterization was done. Both patients and controls filled out a validated Spanish translation of the Fibromyalgia Impact Questionnaire (FIQ) [8].

This research complies with the Helsinki Declaration. Informed consent was obtained from all participants; and the Human Research Committee of the National Institute of Cardiology of Mexico approved the study. This group of subjects is the same reported in our previous genetic studies in FM looking for polymorphisms of the adrenergic receptor [9] and catecholamine-degrading enzyme (COMT) [10]. In the present investigation; we were unable to include samples from Spanish individuals; because there was no more DNA material available.

### Genotyping

Genomic DNA from whole blood containing EDTA was extracted by standard techniques [11]. Ten different single-nucleotide polymorphisms (SNPs) were studied (rs4371369; rs4387806; rs4453709; rs4597545; rs6746030; rs6754031; rs7607967; rs12620053; rs12994338; and rs13017637). SNPs were analyzed by 5' exonuclease TaqMan assay on a 7900HT Fast real-time PCR system according to manufacturer's instructions (Applied Biosystems; Foster City; CA). The National Center for Biotechnology Information (Bethesda; MD) SNP database was used to assign SNP numbers.

### Statistical analysis

Statistical analysis was carried out with Stata 8.0 software for Windows (StataCorp; College Station; TX). A chi-square test was used to evaluate Hardy-Weinberg equilibrium for each polymorphism. In the exploratory analysis; numeric data showed a non-normal (non-Gaussian) distribution (*P *> 0.05 by Shapiro-Wilk statistics); therefore; data were measured as median and interquartile range. The Kruskal-Wallis test was used to compare visual analog scale (VAS) scores on the FIQ with SNPs and genotypes. Proportions of patients and controls per SNP were estimated and compared using chi square or Fisher's exact tests; as required; and presented as absolute frequencies and proportions. Statistical significance was set at an alpha level of less than or equal to 0.05. Pair wise linkage disequilibrium (LD; D') estimations between polymorphisms and haplotype reconstruction were performed with Haploview version 3:32 (Broad Institute of Massachusetts Institute of Technology and Harvard University; Cambridge; MA).

## Results

Table 1 summarizes the baseline characteristics of the studied individuals and genotype distribution of all SCN9A SNPs studied. The observed and expected frequencies of the different SNPs were in Hardy-Weinberg equilibrium. When polymorphisms were analyzed; a different distribution of the rs6754031 SNP was observed in patients when compared to healthy controls (*P *= 0.036) mostly due to an absence of the GG genotype in controls. Correlation analysis between SNPs and total FIQ score showed that patients with the rs6754031 GG genotype had higher total FIQ scores (median = 80; percentile 25/75 = 69/88) than patients with the GT genotype (median = 63; percentile 25/75 = 58/73; *P *= 0.002) or the TT genotype (median = 71; percentile 25/75 = 64/77; *P *= 0.001) (Table 2).

**Table 1:** Demographic data and comparison of cases and controls

		Cases	Controls	
Age, mean ± SD years		44 ± 12	43 ± 12	
FIQ score (0-100), mean ± SD		68 ± 12	03 ± 03	
Genotype		n (%)	n (%)	*P*
rs 7607967	AA	35 (46)	20 (43)	NS
	AG	28 (37)	22 (48)	
	GG	13 (17)	4 (9)	

rs 12994338	CC	47 (63)	25 (52)	NS
	CT	25 (33)	19 (40)	
	TT	3 (4)	4 (8)	

rs 4371369	AA	36 (47)	15 (32)	NS
	AG	29 (38)	25 (53)	
	GG	12 (15)	7 (15)	

rs 4453709	AA	13 (16)	8 (17)	NS
	AT	32 (42)	20 (43)	
	TT	32 (42)	19 (40)	

rs 4597545	CC	21 (27)	16 (33)	NS
	CG	42 (55)	18 (38)	
	GG	14 (18)	14 (29)	

rs 4387806	CC	36 (47)	20 (42)	NS
	CT	35 (46)	19 (39)	
	TT	5 (7)	9 (19)	

rs 6754031	GG	8 (11)	0	0.036**
	GT	24 (33)	15 (31)	
	TT	41 (56)	33 (69)	

rs 12620053	AA	34 (47)	17 (35)	NS
	AC	27 (38)	21 (44)	
	CC	11 (15)	10 (21)	

rs 13017637	CC	36 (50)	28 (58)	NS
	CT	25 (34)	18 (38)	
	TT	11 (15)	2 (4)	

rs 6746030	AA	1 (1)	0	NS
	AG	12 (17)	6 (13)	
	GG	57 (82)	40 (87)	

Proportions of genotype in each SNP

** Fisher's exact test

Different numbers of individuals were analyzed for each polymorphism

**Table 2:** Fibromyalgia Impact Questionnaire score in patients with rs6754031

Genotype	Percentile 25	Median	Percentile 75	*P *value
GG	69.74	80.38	88.74	0.039
GT	58.55	63.29	77.53	
TT	64.23	71.25	77.00	

LD analysis among nine SNP markers. showed that six of them (rs4597545; rs4387806; rs6754031; rs12620053; rs13017637; rs12994338) had strong LD. One SNP (rs6746030) was not studied because too few individuals were determined for this variant. In light of the strong LD; we analyzed the most frequent haplotypes in patients and healthy controls to determine whether some of these haplotypes could be associated with the risk of developing FM. The distribution of the different haplotypes was similar in patients and healthy controls (data not shown).

## Discussion

This group of Mexican patients suffering from FM displayed different distribution of SCN9A rs6754031 genotypes when compared to ethnic; age; and gender matched controls. The difference was due mostly to the absence of the rs6754031 GG genotype in controls. Additionally; in this group of FM patients; the rs6754031 GG genotype is associated with severe disease; as assessed by the FIQ. To our knowledge there is no prior research linking rs6754031 with any particular illness. Although no causality could be derived from the present investigation; these preliminary results raise the possibility that some patients with severe FM may have a DRG sodium channelopathy.

We are not aware of previous studies looking at SCN9A sodium channels gene variation in FM. However; Reimann et al. studied several SCN9A polymorphisms in patients with different painful syndromes; including osteoarthritis; sciatica; amputee phantom pain; spine surgery and pancreatitis. They found a link of rs6746030 with pain intensity. They did not study the SNP rs6754031 that we found associated to FM severity [6]. On the other hand; in our study; rs6746030 did not differentiate FM patients from controls. Evidence emerging from these two studies suggests that in different painful conditions; including FM; pain perception may be linked to dorsal root ganglia/sympathetic ganglia sodium channels gene variations. There may be different predisposing polymorphisms for different ethnic groups. In view of the fact that SCN9A Nav1.7 gene encodes sodium channels located preferentially in dorsal root ganglia and sympathetic ganglia; our results provide additional evidence to the concept that FM may be a sympathetically maintained neuropathic pain syndrome [12]. Nav1.7 mutations induce electrical hyperactivity of sensory neurons in DRG; and; at the same time; produce hypo-reactivity of sympathetic ganglia neurons. This paradoxical behavior is also seen in FM. Generalized pain coexists with sympathetic hypo-reactivity to different types of stressors [13].

After the submission of this manuscript Faber et al. published a gain of function SCN9A mutation associated to idiopathic small fiber neuropathy. This novel finding points to a broader role of Na(V) 1.7 mutations in painful neurological disease than previously considered from studies on rare genetic syndromes [14].

Our previous genetic studies in this group of patients found an association of two catechol-o-methyl transferase (COMT) enzyme gene polymorphisms with FIQ subscales. COMT rs6269 was associated with pain and fatigue; and COMT rs165599 with morning stiffness and disability [9]. Another study in this same group looking at adrenergic receptor (AR) gene polymorphisms disclosed an association between beta 2 adrenergic receptor AC haplotype and the presence of FM. In addition; patients with AR rs574584 GG genotype presented the highest FIQ score as compared with patients with other AR rs574584 genotypes. AR SNP rs574584 was associated with FIQ morning stiffness and with FIQ tiredness upon awakening [10]. The results of these studies suggest a complex polygenic genetic predisposition for the development of FM. These three elements (catecholamines; adrenergic receptors; and sodium channels) could theoretically interact in the induction of FM pain.

We recognize limitations in this pilot study. The sample size is not large enough to withstand multiple comparisons of common gene polymorphisms; therefore a type II statistical error is possible. Nevertheless; the following two findings favor a real linkage between rs6754031 GG genotype and Mexican FM patients: 1) rs6754031 polymorphism was significantly different in both groups mostly due to an absence of the GG genotype in controls. 2) In the patients group the same rs6754031 GG genotype was associated to a severe disease. Studies in other ethnic groups with a larger sample size are needed to verify or amend these preliminary observations.

Another limitation of our study is the lack of rs6754031 functional profiling. Techniques such as patch methods or functional expression assays are beyond our capabilities. So; at the present time; the link between SCN9A polymorphism and severe FM is just associative. Nevertheless; our findings raise the possibility that DRG could be an important element in the pathogenesis of FM; a site in which physical; emotional; or infective stimuli could be converted into chronic pain [15]. Genetic functional profiling [3,7,14]; advanced imaging techniques [16] or histology in the recently described FM animal model [17] could test this novel proposal

The results of the present investigation may have potential therapeutic implications. If future investigations confirm the Nav1.7 sodium channels dysfunction in FM; then sodium channel blockers may become a therapeutic option. Preliminary evidence supports this contention. Lignocaine; a non-specific sodium channel blocker; appears to reduce FM pain [18]. Duloxetine; another drug used in FM; blocks Nav1.7 sodium channels in a state-dependent manner [19]. However; current channel blockers are weak and non-specific. Selective tetrodotoxin-resistant channel blockers are being developed and may in the future constitute a therapeutic option for FM pain (1).

## Conclusions

Our results show that; in this group of Mexican women; there is an association of the rs6754031 polymorphism with the risk of developing FM; as well as with the FIQ score. This association raises the possibility that some patients with severe FM may have a DRG sodium channelopathy.

## Abbreviations

FM: Fibromyalgia; DRG: Dorsal root ganglia; FIQ: Fibromyalgia Impact Questionnaire; SNP: Single nucleotide polymorphism; COMT: Catechol o methyl transferase; VAS: Visual analogue scale; AR: Adrenergic receptor

## Competing interests

The authors declare that they have no competing interests.

## Authors' contributions

GVA; EAL and VMF carried out the genetic studies. AM and AG recruited patients and were involved in the clinical part of the investigation. MV performed the statistical analysis. All authors read and approved the final manuscript. MML conceived the study; participated in its design and coordination and helped to draft the manuscript. All authors read and approved the final manuscript.

### Funding

We gratefully recognize the financial support of the American Fibromyalgia Syndrome Association.

## Pre-publication history

The pre-publication history for this paper can be accessed here:

http://www.biomedcentral.com/1471-2474/13/23/prepub
